# Supplementary material for: The hair cell analysis toolbox is a precise and fully automated pipeline for whole cochlea hair cell quantification
Source: PLoS Biol. 2023 Mar 22;21(3):e3002041. doi: 10.1371/journal.pbio.3002041 (PMC10069775; doi:10.1371/journal.pbio.3002041)
Supplement: Attachment — Submitted filename: response_to_review_v3.pdf [file pbio.3002041.s005.pdf]

Dear Editor and Reviewers,

We thank you for the thoughtful comments and suggestions on ways to improve the manuscript. After careful reading of your comments, we have made appropriate changes in the manuscript which we believe improved its overall quality and clarity of the message conveyed. Below you can find our point-by-point responses for each comment.

Editor:

1) Consider a title change to more clearly convey the utility of this tool:

   Thank you for the suggestion. We changed the title to “The Hair Cell Analysis Toolbox is a precise and fully-automated pipeline for whole cochlea hair-cell quantification”.

2) Consider a somewhat pared down abstract, removing some of the background and focusing more on the interesting findings in this work:

   Thank you for the suggestion, we’ve adopted Editor’s version of the abstract with one minor clarification.

3) Please go through your submission and make sure to clearly state the species being referred to in each instance within the results and in the methods.

   We have made appropriate additions in the manuscript text and figure legends.

4) Please provide summary data for the following graphs:
   Fig1G; Fig3C,F,G, Fig4F,G; *Fig5A-C (note that we only need summary data, not raw data), Fig6A,B; Supplemental Fig1B-E;

   We have included multiple CSV files, which contain the necessary data to replicate our figures. Figure 3C is meant to be a schematic representation, hence there is no summary data to provide for it. We have clarified that in the figure legend.

Reviewer 1:

1) Figure 1. Although it's more or less obvious that the paper is about the mouse cochlea, this should be stated in the Methods, including genotype, and tissue source should be identified in each Figure legend including species and age.

   We have made appropriate additions in the manuscript text and figure legends.

2) Figure 1. Scale bars for panels A, B, C? Better still, mark off distance in microns along the entire length of image in Panel A.

   Thank you for the suggestion, we have added distance in microns along the entire lengths of images in panels A, B and C (Figure 1).

3) How are multiple turns flattened to obtain panel A? The mouse cochlea has approximately 2.5 turns in situ. How does the tissue become a single arc? Is this by shaping after dissection? Or is this from the image analysis? If from the analysis, is it possible to illustrate diagrammatically? Not absolutely necessary, but would help the Reader understand the process.

   Our preparation is a neonatal whole mount of a mouse cochlea. These are common across different experiments when working with early postnatal cochlear tissue. At this age, the cochlear epithelium can

be dissected as a single contiguous ribbon, which is then mounted on a coverslip taking the shape shown on Figure 1A. This preparation can be achieved up to ~P7. We have added clarifications to the Figure legend and the methods section.

4) Panel A lists phalloidin to label stereocilia as blue, and anti-myo7A to label hair cell somata as red. While it's not possible to distinguish in panel A, in panel B it appears that the stereociliary bundles are red (ish) and the hair cells bodies are blue (ish). Labels reversed?

Thank you for spotting our mistake! This has now been corrected.

5) Figure 2A. IHCs and OHCs pseudo-colored? Or are these the little squares at low mag??

We apologize for the confusion. The hair cell annotations (the little squares) appear as a strip of yellow line at low magnification. Due to the rendering approach, they appear brighter than the cells themselves. We have adjusted the figure legend to clarify this.

6) Table 1 - # of images constituting what total cochlear length? This would help to evaluate whether each observer found the same hair cell density (i.e., per 100 microns).

Unfortunately, only the imaging data generated in our lab were of entire intact cochlea. All community provided images were of various regions, often between 15-40 IHCs wide, while some were imaged at lower magnification, yielding images that included even more hair cells. We do not, however, have accurate pixel sizes (and other metadata) for most of our community provided data. We agree the hair cell density is a valuable metric, however it might vary significantly based on species or the age of the animal. We do not expect the same hair cell density across all training data.

We do, however, realize the importance of this information for any future reuse of these data. To address this, we are preparing a Data Descriptor publication in which we hope to gather and provide more detail about community provided datasets, likely include their metadata and individual methods sections for each laboratory. The Data Descriptor manuscript will be co-authored by all groups that provided community data and will be accompanied by raw data depositions (along with our manual data annotations) to online data repositories. We hope by releasing all training data and annotations used in this study we will help to expedite this type of work in the future, while also adhering with NIH policy on Data Management and Sharing.

7) Figure 3. "encoded crops are classified into OHC and IHC classes" manually?

The classification of hair cells after detection is performed by the trained neural network as a part of the entire pipeline. This has now been clarified in the figure legend.

8) Lines 168-169 versus lines 174-175. Should these values be the same?
Line 177 - 0.15%, typo?

Thank you for spotting this mistake! This has now been corrected in the text.

9) Table 2 - total cochlear length in images?

Since these images were taken as screenshots of data from publications, most represent small cochlear regions typically presented in figure panels. The cochlear length per image can be roughly estimated by the total number of inner hair cells (across all images from a given publication), which we report in column 4 of Table 2.

10) Have the authors considered analysis for 'subcellular' biomarkers such as pre- and postsynaptic proteins? Some immunolabels have very high signal to noise (e.g., anti-CtBP2, anti-synapsin, anti-Na\K ATPase).

This is a great idea, and it has been discussed and considered in the lab. It is, however, out of scope for this study, and we are aware of other groups currently making great progress on this. We agree that a similar approach could be used for counting hair cell synapses.

Reviewer 2:

1) *Major Point*: This toolbox can accept two-dimensional data (projection images) of immunostained cochleae. Recent advancements in tissue-clearing techniques enabled the acquisition of the intact 3D structure of the cochlea using confocal or two-photon laser scanning microscopy. Such 3D image data require programs different from the toolbox presented in this manuscript and will become more critical in the realistic modeling of sound propagation through the cochlear duct. This point should be clarified in the introduction part.

   Thank you for your suggestion, we have clarified this point in the introduction and discussion sections of the manuscript. Furthermore, we have attempted HCAT detection analysis on maximum projected, tissue cleared cochleae and saw sub-satisfactory results, likely due to the larger pixel size and lack of bundle staining. In the future, our approach may be improved by the addition of such training data.

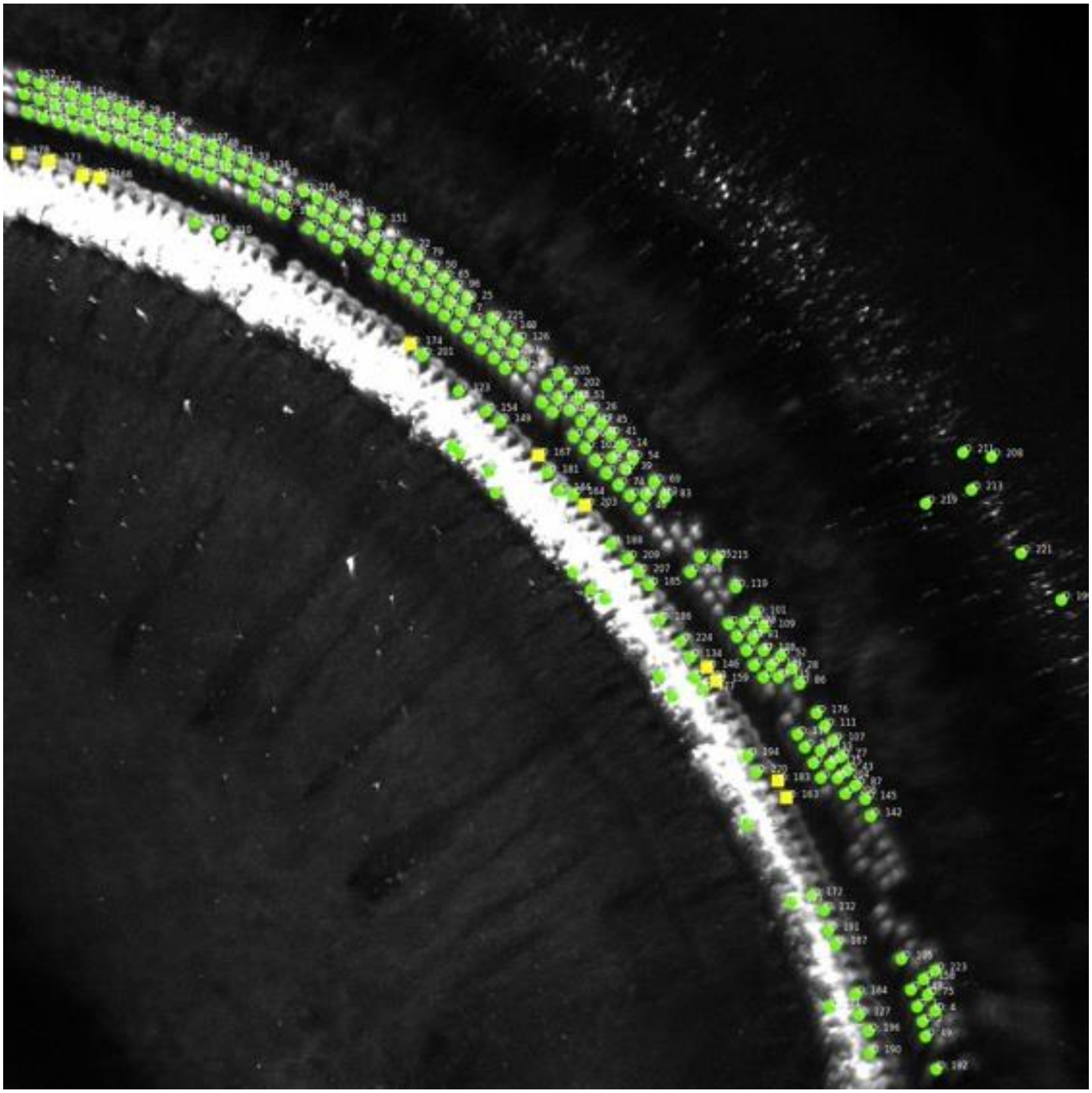

The image above is from (Urata et al. 2019), of a cochlear turn labeled with anti-MYO7a and imaged at ~0.99 um/pixel. Unfortunately, this image contains no bundle stain data.

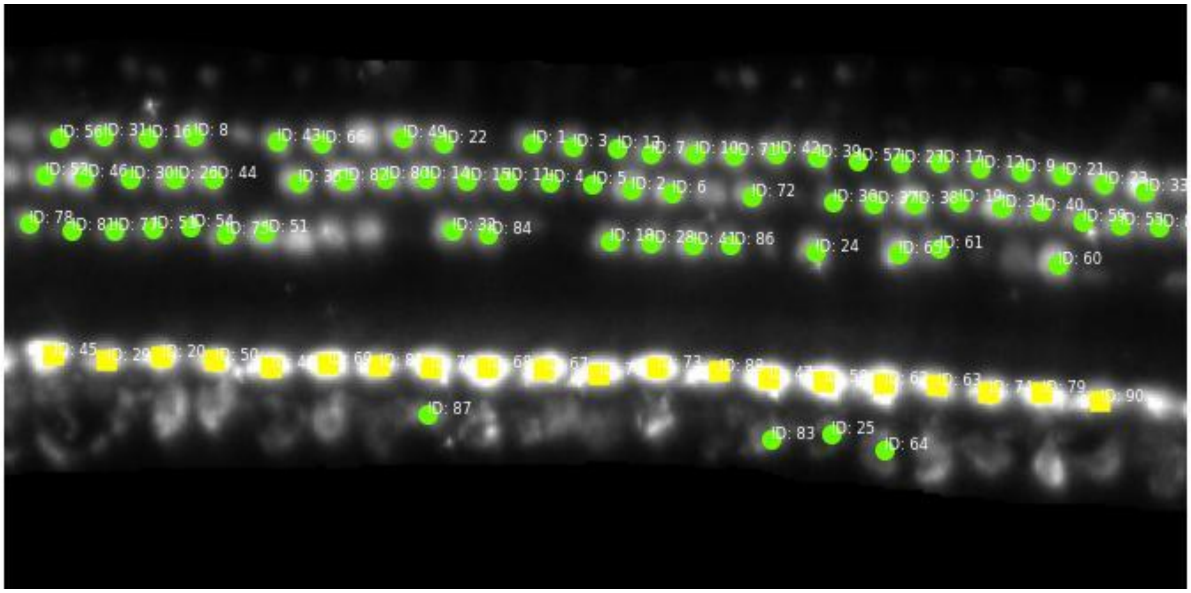

We also attempted the hair cell detection analysis on another tissue cleared dataset (newborn pig cochlea) and saw slightly improved results, especially on IHC detection, likely due to slightly higher resolution imaging (0.65µm/pix). This image was from (Moatti et al. 2020), and also only contains anti-MYO7a fluorescence. In addition, authors 'cleaned' the image by manually removing all non-specific anti-MYO7A signal which would also improve the tool performance by decreasing the false-positive rate.

2) *Major Point*: The toolbox can reliably detect the position of outer and inner hair cells (OHC and IHC)in the middle part of the organ of Corti. However, it may be difficult to identify OHC and IHC at both ends of the structure (apical and basal ends of the organ of Corti). Therefore, information about the reliability of cell detection at the two ends should be presented.

   Thank you for this suggestion, we quantified the detection accuracy along the cochlea at 10% bin size, presented as Figure 4G in the current version of the manuscript.

3) *Major Point*: The samples analyzed in this study mainly came from manually dissected organs of Corti at P5. The dissection of the adult organ of Corti is challenging and, in many cases, results in fragmentation of the structure. Can this protocol be applied to the analysis of the whole cochlear structure from the adult mouse? If this is feasible, please show the examples.

   Thank you for this comment. While it is true that our tool has been primarily designed to be used with early postnatal cochlear samples due to the abundance of their use across the field for various studies involving hair cell quantification, we believe it can also be used for adult cochlear tissue. We have created a new supplemental figure (Suppl. Fig. 3) illustrating this process.

4) *Minor Points*: For the analysis of cochlear pathology, the positions of lost OHCs/IHCs are more important than the remaining OHCs/IHCs. The authors presented the loss of a small number of cells in Figure 4E. However, it is unclear if the toolbox can identify the original locations of lost cells in the cochlea after noise-induced hearing loss, which often shows massive cell loss at the specific locations along the longitudinal axis of the organ of Corti. It is desirable to show the toolbox's ability to find the position of lost cells.

   Unfortunately, HCAT is unable to detect missing cells. This has now been clarified in the manuscript. While it is possible to visually detect individual missing hair cells, especially if an X-shaped scar is visible on the epithelium, we believe it would be hard to even have 3 human observers agree on the number of missing hair cells once the hair cell loss is greater than a few missing cells within an intact epithelium.

5) *Minor Point*: In Table 1, please show the age of the animals.

Thank you, ages have been added across the manuscript.

6) *Minor Point*: In Table 2, please show the extent of cell loss for samples with noise trauma.

   Thank you for this suggestion, we have added a column in Table 2 listing our estimate of hair cell loss.

7) *Minor Point:* In Supplemental Figure 1, the sample No. 4 with the straightened sensory epithelium showed the highest frequency error. The relationship between the sample deformation and the frequency error should be discussed. Is this sample No.4 different from the one mentioned in the text ("due to the threshold settings of the MYO7A channel, causing an error at very low and very high frequencies")?

   That is correct, our Sample No. 5 was the sample referenced in the text. For this sample, the cochlear path length determination was shorter than actual, likely due to a weak myo7a signal in that tissue sample. It is for this reason we have a built-in functionality to manually define curvature points, bypassing the automated approach altogether for applications requiring higher accuracy.

Reviewer 3:

1) They mention in the paper that other markers can be used like Calb and Esp so it would be nice to see a representative example of what that and the ROI would look like using those (or other) markers.

   All data we used to validate come from screenshots from ~20 published manuscripts. We agree with the reviewer that representative examples would strengthen the paper, but this would require a great effort of getting multiple permissions from both the authors and the publishers to allow for re-printing their images in our paper. Therefore, without an agreement with the original publisher, we are unable to show the images in the manuscript. However, below are some examples of images used to generate the graphs presented in Figure 5. Since this response is likely to also be published online should the manuscript be accepted, we will let the editorial staff decide whether it's appropriate to keep the images in the online version of the response file.

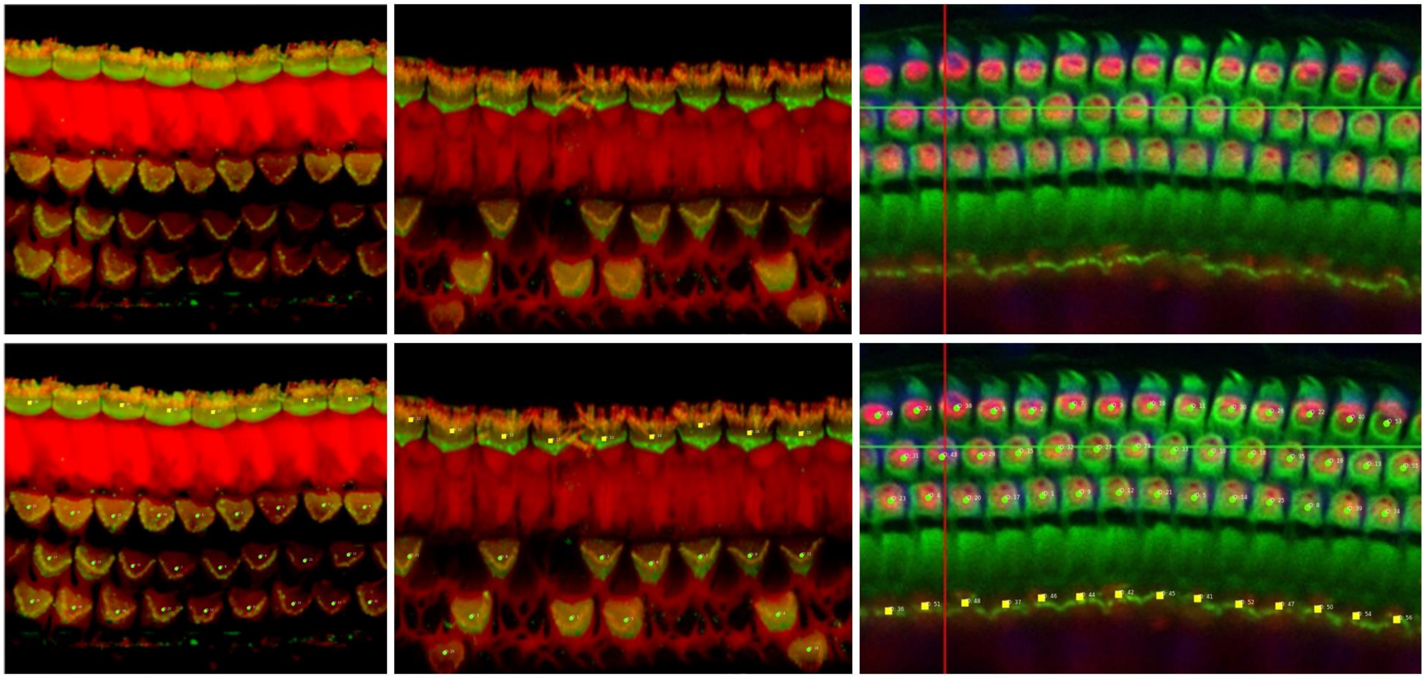

Actin + **Calbindin** *Beurg et al., 2019* **Actin** + **Calbindin** *Beurg et al., 2019* **Actin** + **MYO7A** *Fang et al., 2019*

The images above were stained against Actin and MYO7A, originally from (Beurg et al., 2019) and (Fu et al. 2021)

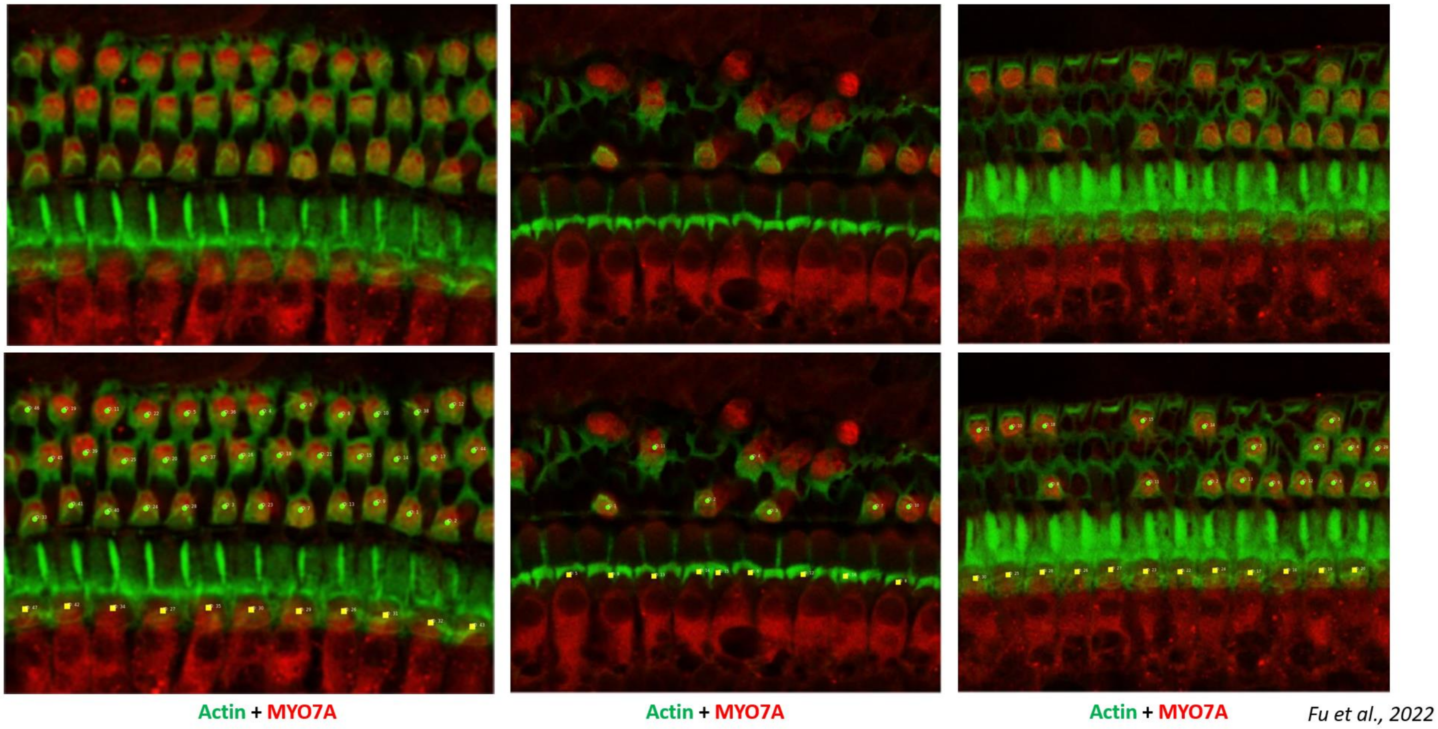

The images above were stained against Actin and MYO7A, originally from (Fu et al. 2021)

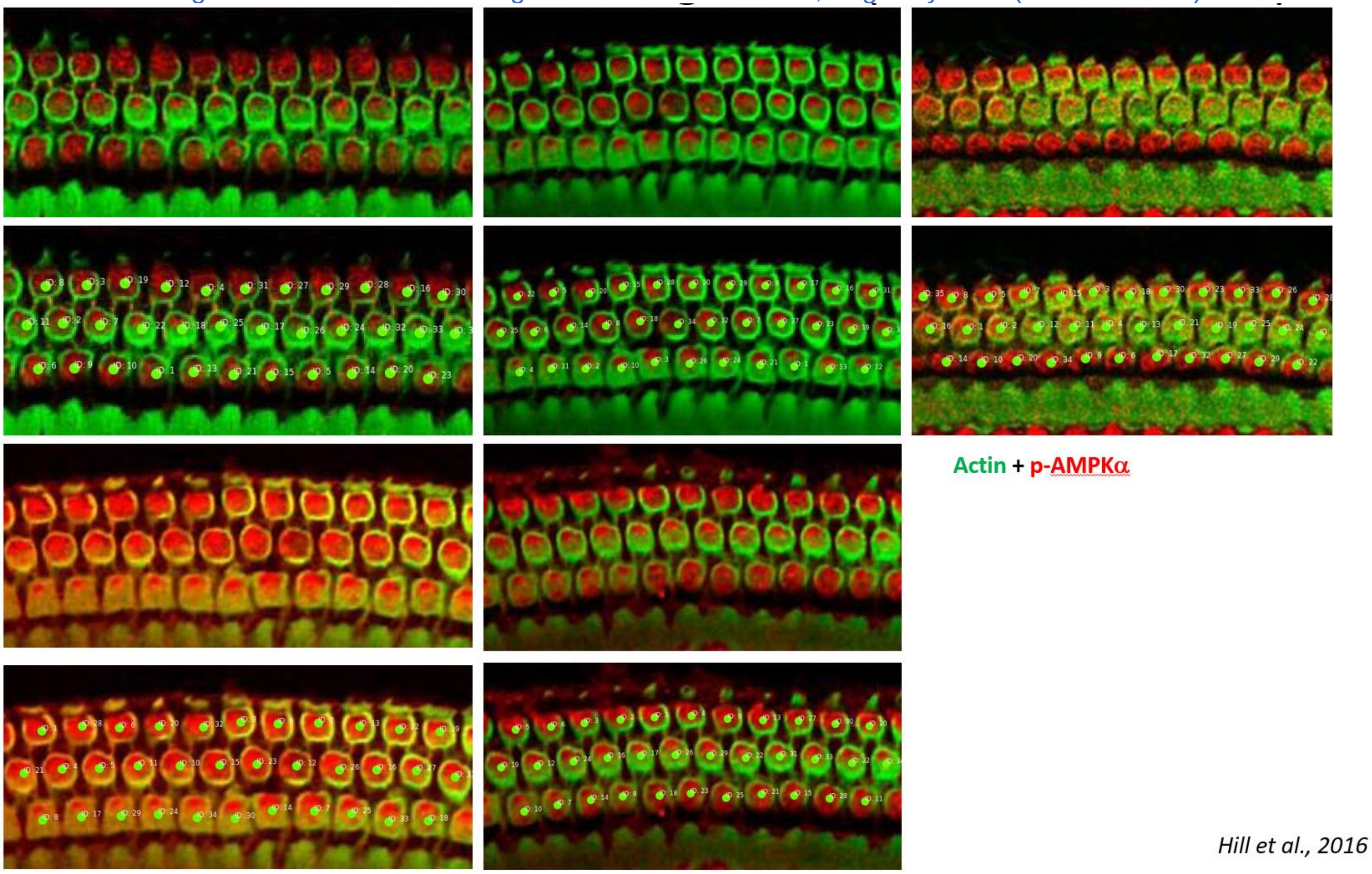

The images above were stained against p-AMPKa and Actin, taken from (Hill et al. 2016)

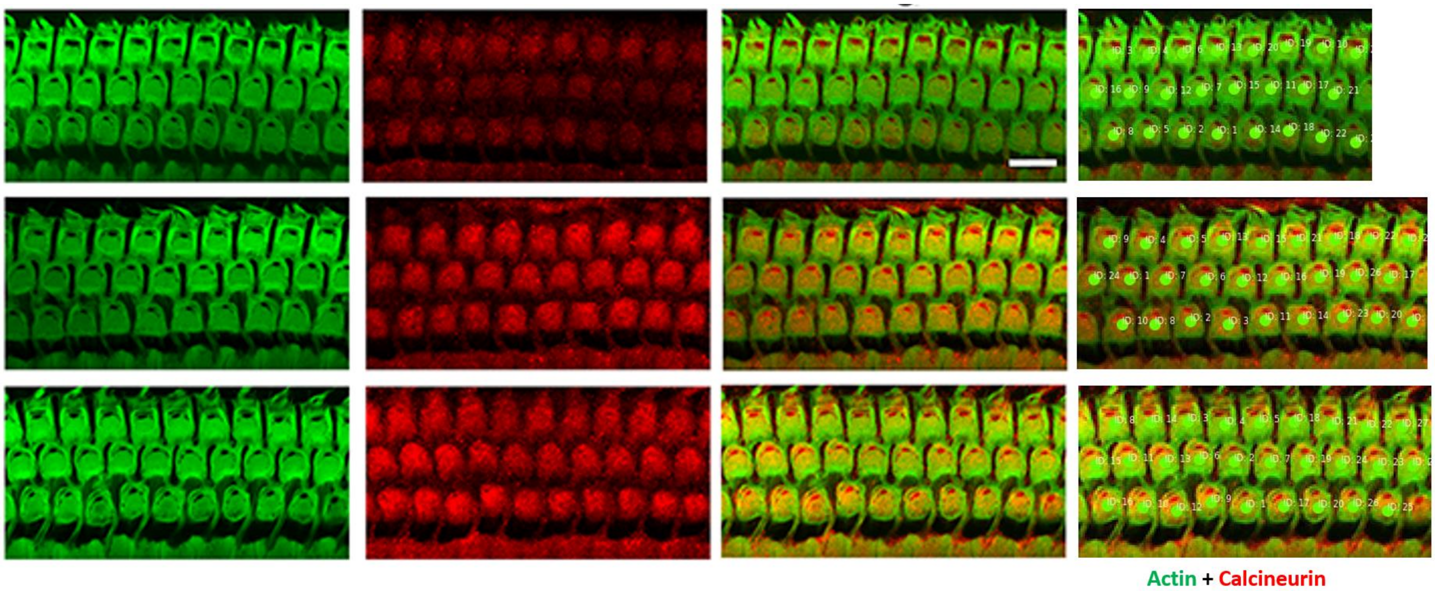

He et al., 2021

The images above were stained against Calcineurin and Actin, from: (He et al. 2021)

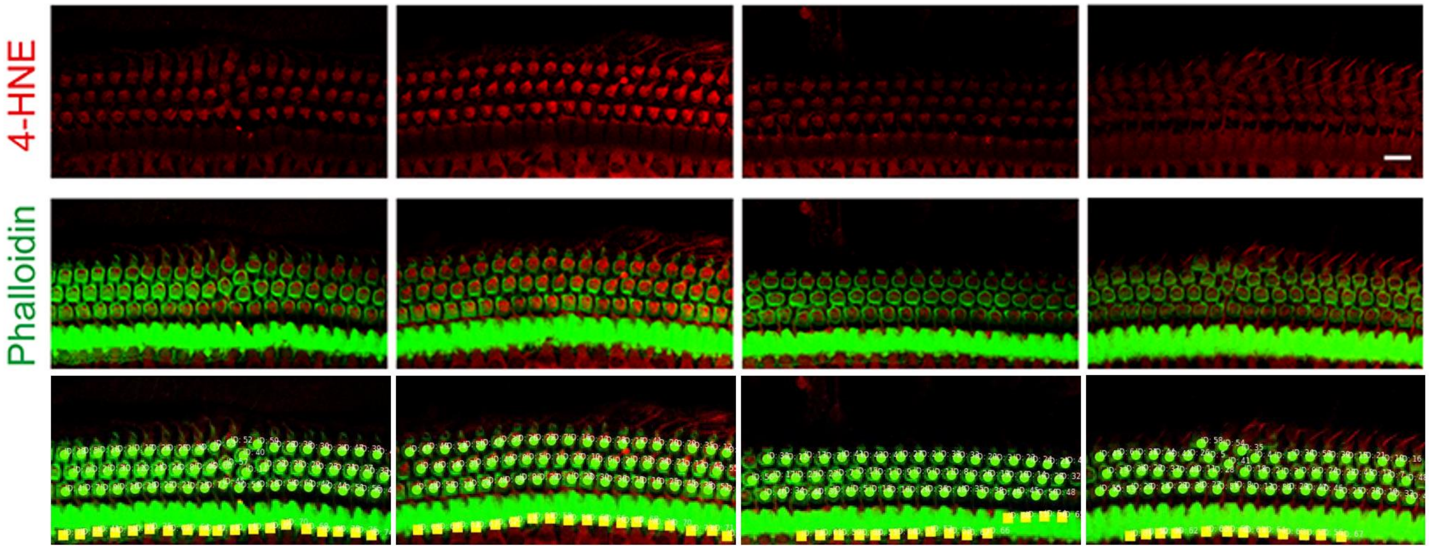

He et al 2021

The images above were stained against 4-HNE and Actin, from: (He et al. 2021)

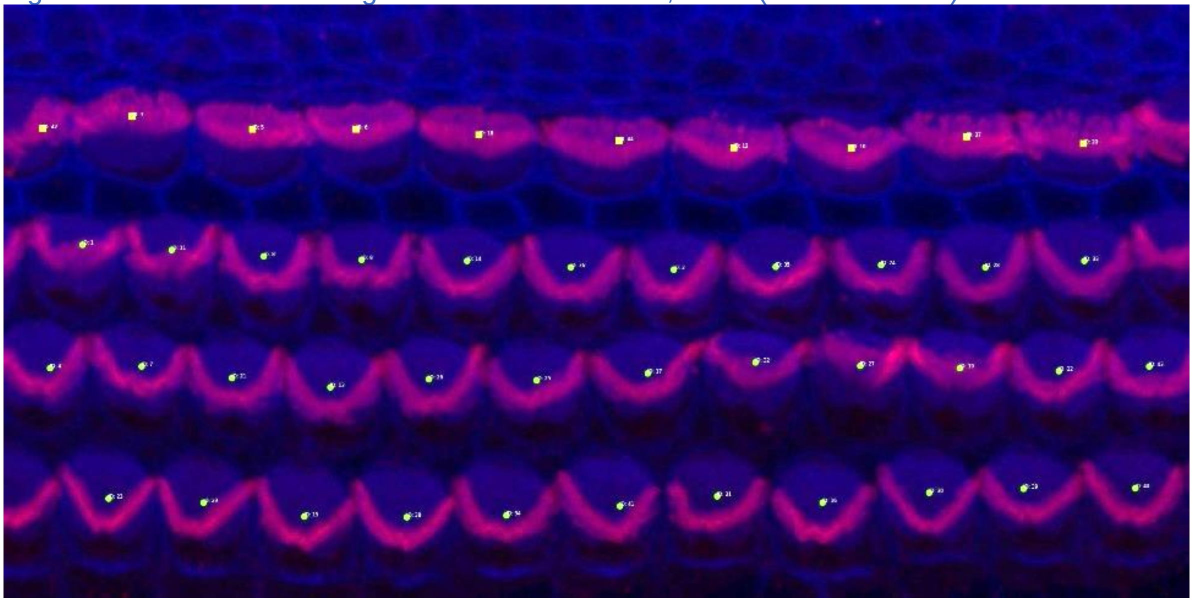

The images above were stained against Espin and Myo7a (Hayashi et al. 2021).

2) Can the method be used to count hair cells without bundles?

Yes, provided there is sufficient clarity in the other marker to distinguish between hair cells. We however believe the stereocilia bundle signal to be a critical signal for our trained model, therefore we expect detection without a bundle stain signal to be of lower accuracy than if it was present. It is however likely to work better in adult cochlear samples as the hair cell bodies do not appear to contact each other as much as in early postnatal cochlear samples.

3) How do they distinguish between inner and outer hair cells. Is it based on phalloidin pattern or localization?

The model performs this action supplemented with a confidence score. The decision is likely based on the shape of inner vs outer hair cells, differences in bundle morphology, and location. Deep learning models are often black boxes, however, therefore we cannot determine with certainty how it makes its decisions on classification.

4) L126 they look at ototoxic induced cochleae from different labs which I think was a good compliment to the study. However, there's no representative images of these and how they were quantified. It would be nice to see how these ROIs were done with damage visually even though they do quantify.

Similar to our response to comment #1, these images are part of another study, and some have been previously published in other manuscripts, and therefore we cannot show these images without obtaining a licensing agreement with the publisher along with the permission from the laboratories that generated these images. Instead, we are pleased to provide representative images from our analysis here:

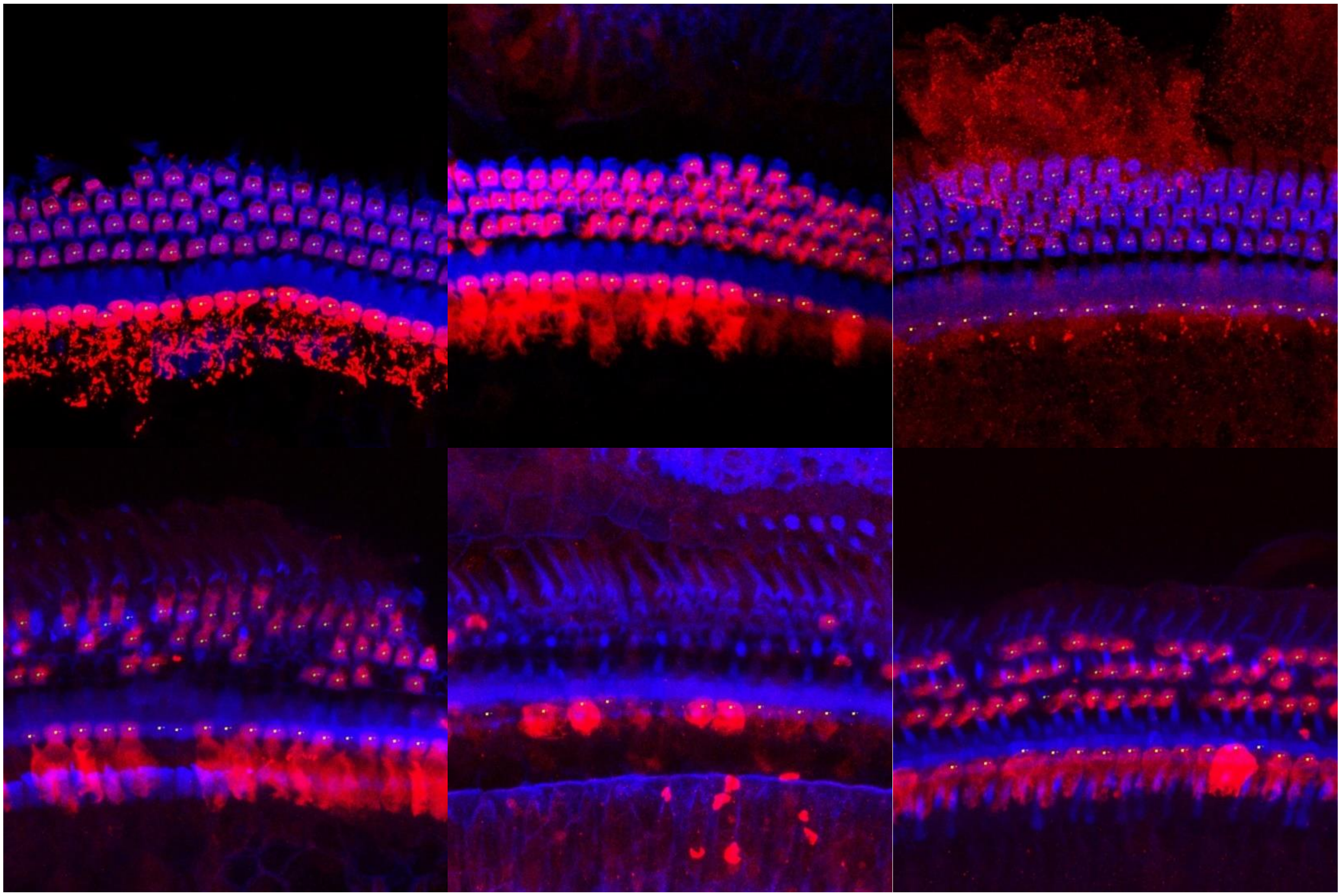

Some representative images from (Gersten et al. 2020) study (using adult mouse cochlea for in-vivo ototoxic drug application) are presented above. The blue channel is phalloidin, while red is anti-MYO7A. These overlaid annotations were generated by the HCAT software. Green dots are automated detections of outer hair cells, while yellow squares are detections of inner hair cells. In all cases, a human observer inspected the data, as well as made the ground truth estimates to generate our results presented in Figure 6.

5) Can the method be applied to a nuclear hair cell stain? E.g. pou4f3?

Our algorithm could, in theory, be applied to any combination of stains provided these stains/markers should be sufficiently informative to distinguish hair cells from non-hair cells. Any stain, or combination of stains, which could correctly identify and distinguish IHCs and OHCs from other cell types, could successfully be used to train a similar detection algorithm. Unfortunately, we were able to locate only very few datasets that would have pou4f3 nuclear hair cell stain, with and without a bundle stain to test the model. We plan on expanding the pool of our training data to further improve the model and are open to including such datasets in the future pool of training data. We expect to receive more community data submissions once this manuscript is published.

To address reviewer's comment, we have performed a detection analysis on a screenshot of Fig1A from (Tong et al. 2015). Please see the original figure, our screenshot, a screenshot of the GUI, and the resulting diagnostic image below.

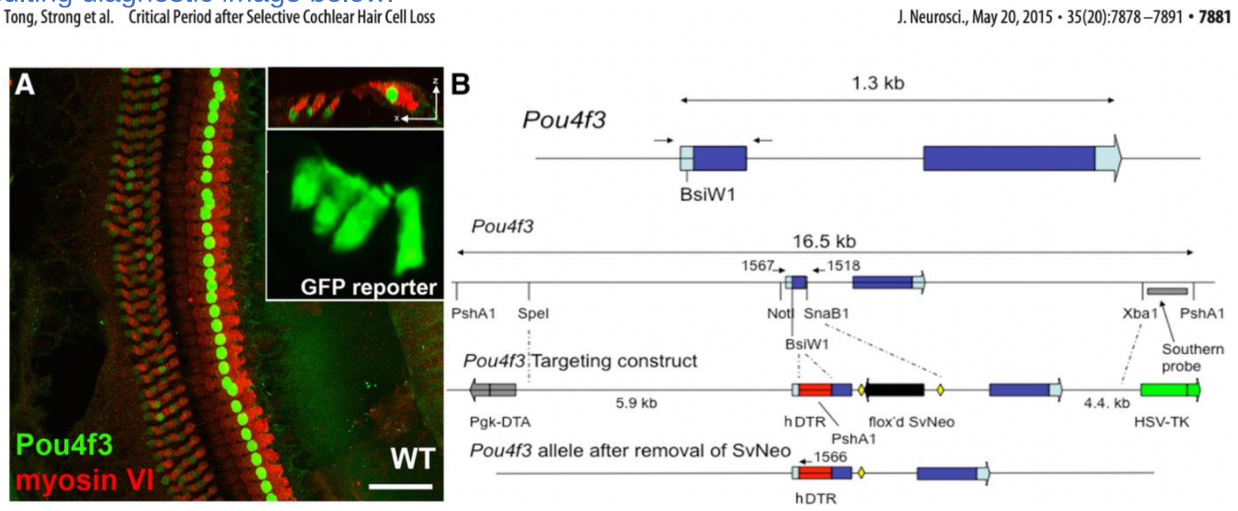

Above is the original Figure 1 from (Tong et al. 2015)

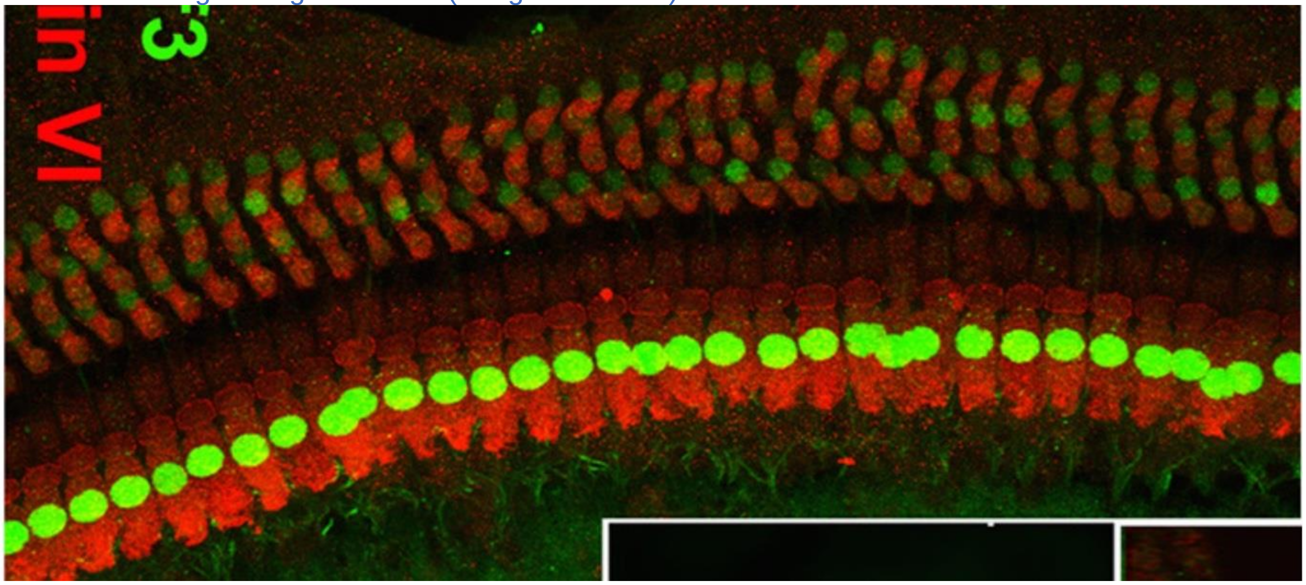

Above is Figure 1A, cropped and rotated

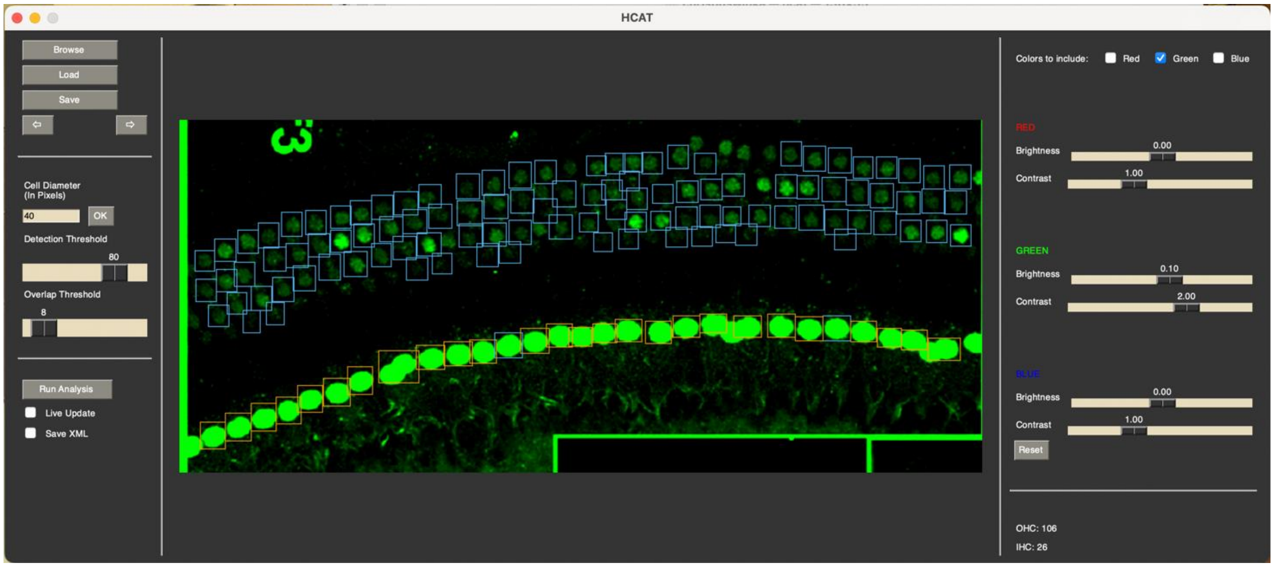

Above is the HCAT GUI capturing the user settings while performing a cell detection analysis utilizing *just* the pou4f3 fluorescence signal.

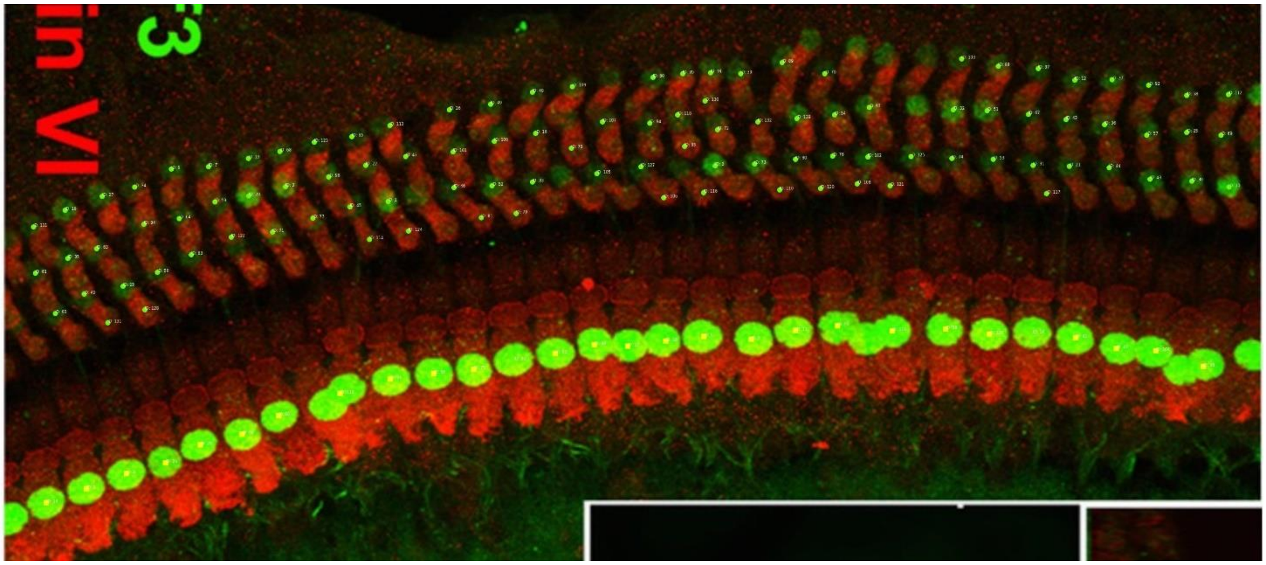

Above is the diagnostic image produced from the resulting analysis.

Similarly, we were able to source an exemplar image that contains two channels of fluorescence information, pou4f3 and phalloidin stain, courtesy of Dr. Bradley Walters' laboratory, resulting in slightly improved performance:

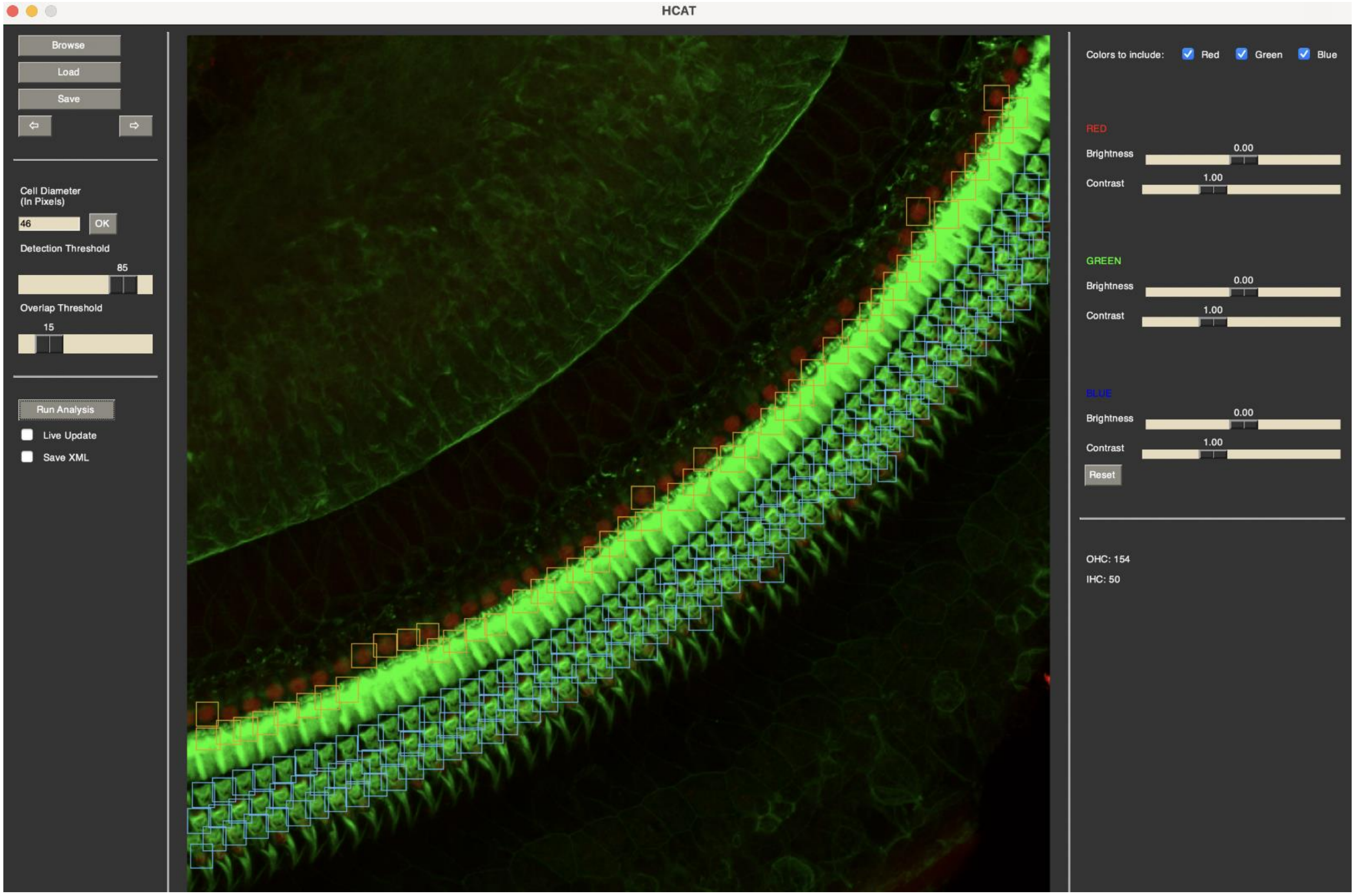

The potential use of pou4f3 has now been suggested within the discussion section of the manuscript.

6) If the location of hair cells is determined by curvature of the cochlear turn, doesn't that depend on how tissues are mounted? If so, that seems problematic.

We apologize for the confusion we may have caused. The curvature is only used to make the Apex vs, Base determination for the cochlear coil, and it can be reassigned manually if needed. The location of each hair cell along the cochlear coil is then determined by the Faster RCNN detection algorithm, while their frequency is assigned by that hair cell’s position along the length of the cochlear coil. The model first calculates a line of best fit through the sensory epithelium, calculates its total length, then assign each cell a frequency based on percentage along that line. We believe this is similar to the commonly used ImageJ plugin, however, it works on a per-cell basis.

7) L214-It would have been nice to have a % accuracy here too, rather than the vaguely worded "sufficient to replicate main finding"

Thank you for this suggestion. While we do not have the hair cell quantification results per image as they were reported with the original study, we have manually analyzed these data and have reported the percentage accuracy in the current version of the manuscript.

8) This seems unnecessary and poorly worded, please remove

We thank the reviewer for this comment. The sentence has been removed.

References:

Fu, Xiaolong, Yachun An, Hongyang Wang, Peipei Li, Jing Lin, Jia Yuan, Rongyu Yue, Yecheng Jin, Jiangang Gao, and Renjie Chai. 2021. 'Deficiency of Klc2 induces low-frequency sensorineural hearing loss in C57BL/6 J mice and human', *Molecular Neurobiology*, 58: 4376-91.

Gersten, Benjamin K., Tracy S. Fitzgerald, Katharine A. Fernandez, and Lisa L. Cunningham. 2020. 'Ototoxicity and Platinum Uptake Following Cyclic Administration of Platinum-Based Chemotherapeutic Agents', *Journal of the Association for Research in Otolaryngology*, 21: 303-21.

Hayashi, Yushi, Hao Chiang, ChunJie Tian, Artur A Indzhykulian, and Albert SB Edge. 2021. 'Norrie disease protein is essential for cochlear hair cell maturation', *Proceedings of the National Academy of Sciences*, 118: e2106369118.

He, Zu-Hong, Song Pan, Hong-Wei Zheng, Qiao-Jun Fang, Kayla Hill, and Su-Hua Sha. 2021. 'Treatment with calcineurin inhibitor FK506 attenuates noise-induced hearing loss', *Frontiers in Cell and Developmental Biology*, 9: 648461.

Hill, Kayla, Hu Yuan, Xianren Wang, and Su-Hua Sha. 2016. 'Noise-induced loss of hair cells and cochlear synaptopathy are mediated by the activation of AMPK', *Journal of Neuroscience*, 36: 7497-510.

Moatti, Adele, Yuheng Cai, Chen Li, Tyler Sattler, Laura Edwards, Jorge Piedrahita, Frances S. Ligler, and Alon Greenbaum. 2020. 'Three-dimensional imaging of intact porcine cochlea using tissue clearing and custom-built light-sheet microscopy', *Biomedical Optics Express*, 11: 6181-96.

Tong, Ling, Melissa K. Strong, Tejbeer Kaur, Jose M. Juiz, Elizabeth C. Oesterle, Clifford Hume, Mark E. Warchol, Richard D. Palmiter, and Edwin W. Rubel. 2015. 'Selective Deletion of Cochlear Hair Cells Causes Rapid Age-Dependent Changes in Spiral Ganglion and Cochlear Nucleus Neurons', *The Journal of neuroscience*, 35: 7878-91.

Urata, Shinji, Tadatsune Iida, Masamichi Yamamoto, Yu Mizushima, Chisato Fujimoto, Yu Matsumoto, Tatsuya Yamasoba, and Shigeo Okabe. 2019. 'Cellular cartography of the organ of Corti based on optical tissue clearing and machine learning', *eLife*, 8.
